# 2-(3,4-Di­chloro­phen­yl)-*N*-methyl-*N*-[2-(pyrrolidin-1-yl)cyclo­hex­yl]acetamide hydro­chloride: (±)-U50,488H

**DOI:** 10.1107/S2414314626006668

**Published:** 2026-06-30

**Authors:** Yoshimi Ichimaru, Masaaki Kurihara

**Affiliations:** a Faculty of Pharmaceutical Sciences Shonan University of Medical Sciences, 16-10 Kamishinano Totsuka-ku Yokohama Kanagawa 244-0806 Japan; Purdue University, USA

**Keywords:** crystal structure, κ-opioid receptor agonist

## Abstract

The crystal structure of the methanol-solvated hydro­chloride salt of the selective κ-opioid receptor agonist (±)-U50,488, namely, 1-{2-[2-(3,4-di­chloro­phen­yl)-*N*-methyl­acetamido]­cyclo­hex­yl}pyrrolidin-1-ium chloride methanol monosolvate, C_19_H_27_Cl_2_N_2_O^+^·Cl^−^·CH_3_OH, is reported. The compound crystallized as a racemate of the (*S*,*S*) and (*R*,*R*) enanti­omers, consistent with the *anti*-selective nucleophilic substitution used in its synthesis. The cyclo­hexane ring adopts a chair conformation with an equatorial-equatorial conformation of its 1,2-substituents. Protonation occurs exclusively at the pyrrolidine nitro­gen atom, and the pyrrolidine ring displays a half-chair conformation twisted on the bond opposite the nitro­gen. In the crystal, the chloride anion forms a hydrogen-bonding network with the protonated pyrrolidine unit, the methanol solvent mol­ecule, and adjacent C—H groups. The crystal packing is further consolidated by C—H⋯O inter­actions involving the amide oxygen atom that form centrosymmetric dimers, alongside inter­molecular π-stacking and Cl⋯O halogen-bonding inter­actions. This study provides the first structural characterization of the hydro­chloride salt form of this widely used pharmacological compound.

## Abstract 2

The crystal structure of the methanol-solvated hydro­chloride salt of the selective κ-opioid receptor agonist (±)-U50,488 is reported. The compound crystallized as a racemate of the (*S*,*S*) and (*R*,*R*) enanti­omers, consistent with the *anti*-selective nucleophilic substitution used in its synthesis. This study provides the first structural characterization of the hydro­chloride salt form of this widely used pharmacological compound.

## Graphical abstract

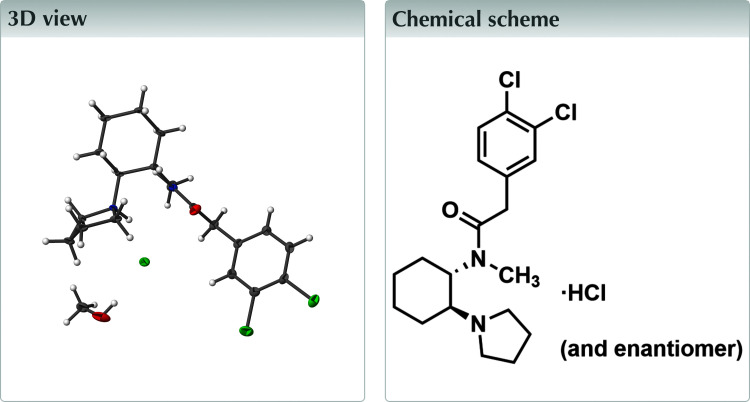

## Structure description

**Figure 1:**
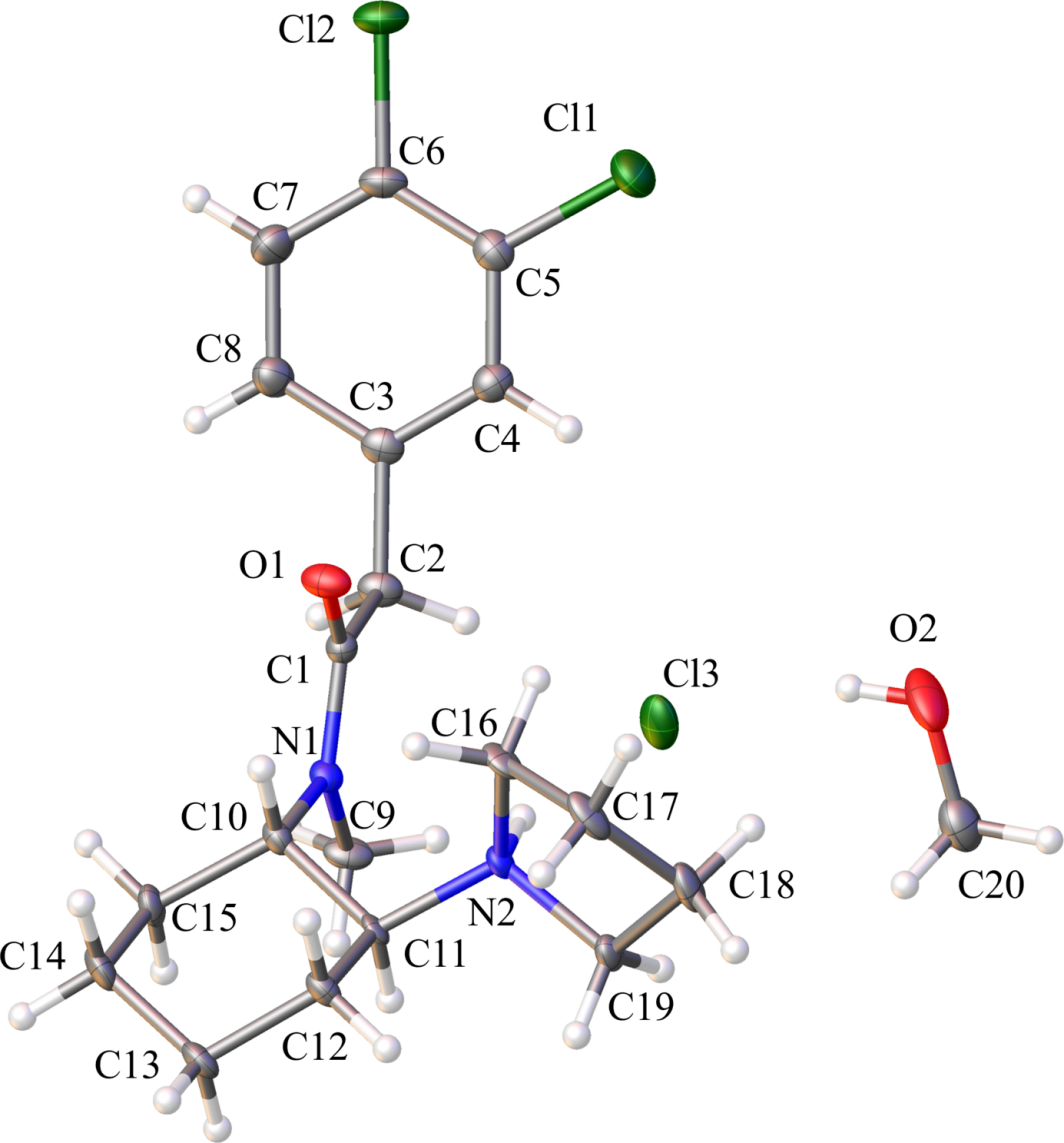
The mol­ecular structure of the title compound with displacement ellipsoids drawn at the 50% probability level.

The title compound (Fig. 1 ▸) is a methanol solvate of the selective κ-opioid receptor agonist commonly known as (±)-U50,488H. ‘U50,488′ was the developmental designation assigned by the Upjohn Company (Szmuszkovicz & Von Voigtlander, 1982 ▸), and it is marketed as ‘U50,488H’ to signify its status as a hydro­chloride salt. To date, the crystal structure of U50,488 has been reported as a methane­sulfonate salt (Doi *et al.*, 1990 ▸). Although U50,488 possesses two chiral centers – potentially yielding four stereoisomers – the standard synthetic route involving the nucleophilic substitution of cyclo­hexene oxide by pyrrolidine selectively produces the *trans*-1,2-cyclo­hexane derivative (Chesis & Welch, 1990 ▸; Kato *et al.*, 2025 ▸). Consequently, the product is obtained as a racemic mixture of the (*S*,*S*) and (*R*,*R*) enanti­omers, while the (*S*,*R*) and (*R*,*S*) isomers are produced in negligible yields. The (*S*,*S*)-enanti­omer corresponds to (−)-U50,488, which exhibits levorotatory properties and possesses a higher affinity for the κ-opioid receptor than its (+)-enanti­omer (Rothman *et al.*, 1989 ▸).

**Figure 2:**
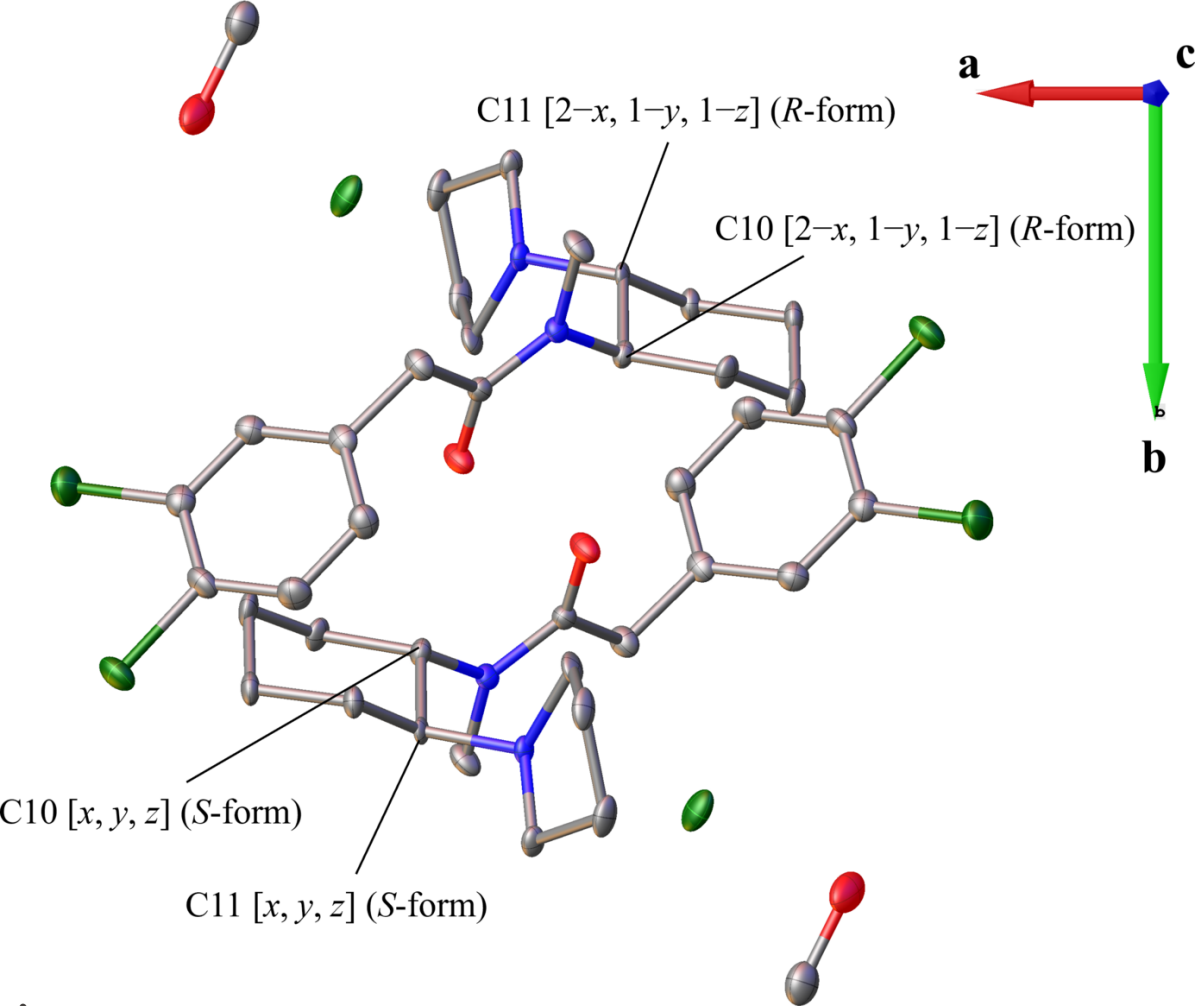
The pair of enanti­omers of the title compound with displacement ellipsoids drawn at the 50% probability level.

**Figure 3:**
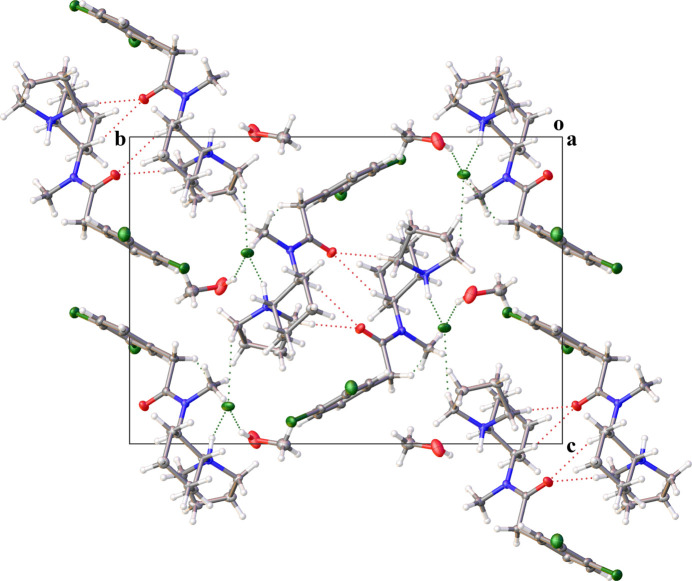
Partial packing diagram viewed along the *a*-axis direction. The green dotted lines represent H⋯Cl inter­actions, and the red dotted lines represent C—H⋯O inter­actions.

The title compound exists as a racemic mixture of the (*S*,*S*) and (*R*,*R*) isomers (Fig. 2 ▸). The cyclo­hexane ring (C10–C15) adopts a chair conformation, as indicated by the Cremer–Pople puckering parameters (Cremer and Pople, 1975 ▸) of *θ* = 2.5 (3)°. The 1,2-substituents on the cyclo­hexane ring adopt an equatorial–equatorial *gauche* conformation: the torsion angle N1—C10—C11—N2 is 53.5(12°. The pyrrolidine ring (N2/C16–C19) adopts a half-chair conformation twisted on the C17—C18 bond, with calculated Cremer–Pople puckering parameters *Q* = 0.413 (3) Å and *φ* = 88.7 (3)°. Due to the significantly reduced basicity of the amide nitro­gen atom (N1), protonation occurs exclusively at the pyrrolidine nitro­gen (N2). The N1—C1 (amide carbon­yl) bond is notably shorter at 1.363 (3) Å, reflecting its partial double-bond character. The distances between the chloride ion (Cl3) and the nitro­gen atoms are 3.5505 (19) Å for Cl3⋯N1 and 3.0775 (19) Å for Cl3⋯N2. The short Cl3⋯N2 inter­atomic distance, which is less than the sum of the van der Waals radii of chlorine and nitro­gen, is attributable to charge-assisted hydrogen-bonding inter­actions arising from the protonation of the pyrrolidine nitro­gen atom. The chloride ion (Cl3) forms a hydrogen bond network with the protonated pyrrolidine, the hydroxyl group of the methanol solvent mol­ecule, and C—H groups (Table 1 ▸). The amide oxygen atom (O1) also acts as a hydrogen-bonding acceptor, inter­acting with nearby C—H groups (Table 1 ▸), contributing to the formation of a centrosymmetric dimer. The torsion angles around the amide group, C10—N1—C1—C2, C9—N1—C1—O1, and N1—C1—C2—C3, are −174.3 (2), −178.5 (2), and 174.6 (2)°, respectively. These angles indicate that the amide bond and its substituents adopt a conformation with minimal steric hindrance. The 3,4-di­chloro­phenyl moiety derived from a carb­oxy­lic acid is involved in π-stacking and halogen-bonding. The plane of the aromatic ring defined by C3–C8 (centroid: *Cg*) has a ring centroid distance of 3.9422 (14) Å and a slippage of 1.690 Å relative to *Cg*^i^ [symmetry code (i): 1 − *x*, 1 − *y*, −*z*]. A halogen bond is formed between the chlorine atom (Cl2) and the methanol oxygen atom (O2^ii^) [3.097 (2) Å; symmetry code (ii): 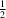 − *x*, −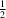 + *y*, 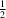 − *z*]. The crystal packing resulting from these inter­molecular inter­actions is illustrated in Fig. 3 ▸.

## Synthesis and crystallization

The title compound was synthesized according to a reported method (Chesis & Welch, 1990 ▸; Kato *et al.*, 2025 ▸). The single crystals suitable for X-ray analysis were obtained by dissolving the compound in a minimum amount of methanol at 298 K followed by slow evaporation.

## Refinement

Crystal data, data collection and structure refinement details are summarized in Table 2 ▸.

## Supplementary Material

Crystal structure: contains datablock(s) I. DOI: 10.1107/S2414314626006668/zl4100sup1.cif

Structure factors: contains datablock(s) I. DOI: 10.1107/S2414314626006668/zl4100Isup2.hkl

Supporting information file. DOI: 10.1107/S2414314626006668/zl4100Isup3.cml

CCDC reference: 2564777

Additional supporting information:  crystallographic information; 3D view; checkCIF report

## Figures and Tables

**Table 1:** Hydrogen-bond geometry (Å, °)

*D*—H⋯*A*	*D*—H	H⋯*A*	*D*⋯*A*	*D*—H⋯*A*
N2—H2⋯Cl3	1.00	2.17	3.0775 (19)	150
C2—H2*A*⋯Cl3	0.99	2.75	3.486 (2)	132
C10—H10⋯O1^i^	1.00	2.61	3.543 (3)	156
C16—H16*B*⋯O1^i^	0.99	2.29	3.274 (3)	170
C19—H19*A*⋯Cl3^ii^	0.99	2.92	3.685 (2)	135
O2—H2*C*⋯Cl3	0.84	2.32	3.103 (2)	155

Symmetry codes: (i) 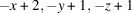; (ii) 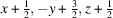.

**Table 2:** Experimental details

Crystal data	
Chemical formula	C_19_H_27_Cl_2_N_2_O^+^·Cl^−^·CH_4_O
*M*_r_	437.82
Crystal system, space group	Monoclinic, *P*2_1_/*n*
Temperature (K)	100
*a*, *b*, *c* (Å)	8.7729 (2), 18.5639 (3), 13.6318 (3)
β (°)	105.022 (2)
*V* (Å^3^)	2144.20 (8)
*Z*	4
Radiation type	Cu *K*α
μ (mm^−1^)	4.01
Crystal size (mm)	0.41 × 0.28 × 0.12

Data collection	
Diffractometer	XtaLAB Synergy, Single source at home/near, HyPix-Bantam
Absorption correction	Multi-scan (*CrysAlis PRO*; Rigaku OD 2025 ▸)
*T*_min_, *T*_max_	0.266, 1.000
No. of measured, independent and observed [*I* > 2σ(*I*)] reflections	23023, 3919, 3518
*R*_int_	0.161
(sin θ/λ)_max_ (Å^−1^)	0.603

Refinement	
*R*[*F*^2^ > 2σ(*F*^2^)], *wR*(*F*^2^), *S*	0.071, 0.197, 1.05
No. of reflections	3919
No. of parameters	247
H-atom treatment	H-atom parameters constrained
Δρ_max_, Δρ_min_ (e Å^−3^)	0.71, −0.93

Computer programs: *CrysAlis PRO* (Rigaku OD, 2025 ▸), *SHELXT* (Sheldrick, 2015*a* ▸), *SHELXL2019/3* (Sheldrick, 2015*b* ▸) and *OLEX2* (Dolomanov *et al.*, 2009 ▸).

## full crystallographic data

### 1-{2-[2-(3,4-Dichlorophenyl)-N-methylacetamido]cyclohexyl}pyrrolidin-1-ium chloride methanol monosolvate Crystal data

**Table:**

C_19_H_27_Cl_2_N_2_O^+^·Cl^−^·CH_4_O	*F*(000) = 928
*M**_r_* = 437.82	*D*_x_ = 1.356 Mg m^−^^3^
Monoclinic, *P*2_1_/*n*	Cu *K*α radiation, λ = 1.54184 Å
*a* = 8.7729 (2) Å	Cell parameters from 12899 reflections
*b* = 18.5639 (3) Å	θ = 4.1–68.2°
*c* = 13.6318 (3) Å	µ = 4.01 mm^−^^1^
β = 105.022 (2)°	*T* = 100 K
*V* = 2144.20 (8) Å^3^	Block, colourless
*Z* = 4	0.41 × 0.28 × 0.12 mm

### 1-{2-[2-(3,4-Dichlorophenyl)-N-methylacetamido]cyclohexyl}pyrrolidin-1-ium chloride methanol monosolvate Data collection

**Table:**

XtaLAB Synergy, Single source at home/near, HyPix-Bantam diffractometer	3518 reflections with *I* > 2σ(*I*)
Detector resolution: 10.0000 pixels mm^-1^	*R*_int_ = 0.161
ω scans	θ_max_ = 68.5°, θ_min_ = 4.1°
Absorption correction: multi-scan (CrysAlisPro; Rigaku OD 2025)	*h* = −10→10
*T*_min_ = 0.266, *T*_max_ = 1.000	*k* = −22→22
23023 measured reflections	*l* = −16→16
3919 independent reflections	

### 1-{2-[2-(3,4-Dichlorophenyl)-N-methylacetamido]cyclohexyl}pyrrolidin-1-ium chloride methanol monosolvate Refinement

**Table:**

Refinement on *F*^2^	Primary atom site location: dual
Least-squares matrix: full	Secondary atom site location: difference Fourier map
*R*[*F*^2^ > 2σ(*F*^2^)] = 0.071	Hydrogen site location: inferred from neighbouring sites
*wR*(*F*^2^) = 0.197	H-atom parameters constrained
*S* = 1.05	*w* = 1/[σ^2^(*F*_o_^2^) + (0.1498*P*)^2^] where *P* = (*F*_o_^2^ + 2*F*_c_^2^)/3
3919 reflections	(Δ/σ)_max_ = 0.001
247 parameters	Δρ_max_ = 0.71 e Å^−^^3^
0 restraints	Δρ_min_ = −0.93 e Å^−^^3^

### 1-{2-[2-(3,4-Dichlorophenyl)-N-methylacetamido]cyclohexyl}pyrrolidin-1-ium chloride methanol monosolvate Special details

**Table:**

Geometry. All esds (except the esd in the dihedral angle between two l.s. planes) are estimated using the full covariance matrix. The cell esds are taken into account individually in the estimation of esds in distances, angles and torsion angles; correlations between esds in cell parameters are only used when they are defined by crystal symmetry. An approximate (isotropic) treatment of cell esds is used for estimating esds involving l.s. planes.
Refinement. H atoms were positioned geometrically and constrained to ride on their parent atoms, with carbon hydrogen bond distances of 0.95 Å for aromatic C—H, 1.00, 0.99 and 0.98 Å for aliphatic C—H, CH_2_ and CH_3_, 1.00 Å for N—H and 0.84 Å for OH moieties, respectively. Methyl and hydroxyl H atoms were allowed to rotate but not to tip to best fit the experimental electron density. *U*_iso_(H) values were set to a multiple of *U*_eq_(O/C/N) with 1.5 for CH_3_ and OH, and 1.2 for C—H, CH_2_ and N—H units, respectively.

### 1-{2-[2-(3,4-Dichlorophenyl)-N-methylacetamido]cyclohexyl}pyrrolidin-1-ium chloride methanol monosolvate Fractional atomic coordinates and isotropic or equivalent isotropic displacement parameters (Å^2^)

**Table:**

	*x*	*y*	*z*	*U*_iso_*/*U*_eq_	
Cl1	0.25998 (8)	0.51248 (4)	0.18314 (6)	0.0315 (3)	
Cl2	0.34226 (7)	0.37367 (3)	0.07217 (5)	0.0256 (2)	
Cl3	0.71467 (7)	0.72770 (3)	0.37791 (4)	0.0259 (2)	
O1	0.8975 (2)	0.53307 (9)	0.37621 (13)	0.0208 (4)	
N1	1.0566 (2)	0.62855 (9)	0.36832 (15)	0.0135 (4)	
N2	0.9968 (2)	0.68267 (10)	0.55323 (14)	0.0126 (4)	
H2	0.931549	0.690415	0.482139	0.015*	
C1	0.9314 (3)	0.58443 (11)	0.32909 (17)	0.0138 (5)	
C2	0.8339 (3)	0.60260 (12)	0.22206 (18)	0.0183 (5)	
H2A	0.783263	0.650196	0.222737	0.022*	
H2B	0.904466	0.605784	0.176132	0.022*	
C3	0.7089 (3)	0.54694 (12)	0.18224 (17)	0.0169 (5)	
C4	0.5595 (3)	0.55431 (13)	0.19764 (18)	0.0186 (5)	
H4	0.535040	0.595382	0.232240	0.022*	
C5	0.4456 (3)	0.50177 (13)	0.16259 (19)	0.0195 (5)	
C6	0.4811 (3)	0.44110 (13)	0.11320 (18)	0.0198 (5)	
C7	0.6304 (3)	0.43311 (13)	0.0983 (2)	0.0235 (6)	
H7	0.655781	0.391391	0.065389	0.028*	
C8	0.7426 (3)	0.48643 (13)	0.13171 (19)	0.0214 (5)	
H8	0.844125	0.481478	0.119847	0.026*	
C9	1.0923 (3)	0.69030 (13)	0.31167 (18)	0.0196 (5)	
H9A	1.182676	0.716593	0.353736	0.029*	
H9B	1.000369	0.722303	0.293644	0.029*	
H9C	1.117651	0.673449	0.249634	0.029*	
C10	1.1646 (3)	0.61037 (12)	0.46710 (18)	0.0139 (5)	
H10	1.126337	0.564529	0.491028	0.017*	
C11	1.1637 (2)	0.66856 (11)	0.54710 (17)	0.0120 (5)	
H11	1.205183	0.714079	0.524470	0.014*	
C12	1.2738 (3)	0.64729 (12)	0.64974 (18)	0.0159 (5)	
H12A	1.273195	0.685300	0.700574	0.019*	
H12B	1.236782	0.601850	0.673823	0.019*	
C13	1.4414 (3)	0.63738 (13)	0.63785 (19)	0.0179 (5)	
H13A	1.478661	0.683180	0.614975	0.021*	
H13B	1.513394	0.624491	0.704281	0.021*	
C14	1.4445 (3)	0.57836 (14)	0.5609 (2)	0.0219 (6)	
H14A	1.412137	0.532024	0.585366	0.026*	
H14B	1.553129	0.572787	0.553452	0.026*	
C15	1.3325 (3)	0.59729 (12)	0.45804 (19)	0.0179 (5)	
H15A	1.371233	0.641097	0.430812	0.022*	
H15B	1.331622	0.557414	0.409668	0.022*	
C16	0.9187 (3)	0.62200 (12)	0.59818 (19)	0.0180 (5)	
H16A	0.814673	0.609496	0.552426	0.022*	
H16B	0.986002	0.578427	0.609943	0.022*	
C17	0.9002 (3)	0.65241 (14)	0.69791 (19)	0.0221 (5)	
H17A	0.998214	0.646247	0.752864	0.027*	
H17B	0.811734	0.628975	0.718283	0.027*	
C18	0.8657 (3)	0.73197 (14)	0.6734 (2)	0.0224 (6)	
H18A	0.755600	0.739218	0.632876	0.027*	
H18B	0.884580	0.761229	0.736169	0.027*	
C19	0.9823 (3)	0.75050 (12)	0.61262 (18)	0.0157 (5)	
H19A	1.085729	0.763921	0.658225	0.019*	
H19B	0.942900	0.791172	0.565949	0.019*	
O2	0.4718 (2)	0.78838 (11)	0.48632 (19)	0.0388 (6)	
H2C	0.528067	0.759651	0.463420	0.058*	
C20	0.5452 (3)	0.85588 (16)	0.5019 (2)	0.0304 (6)	
H20A	0.651834	0.850649	0.546605	0.046*	
H20B	0.483688	0.888338	0.533538	0.046*	
H20C	0.551417	0.875904	0.436562	0.046*	

### 1-{2-[2-(3,4-Dichlorophenyl)-N-methylacetamido]cyclohexyl}pyrrolidin-1-ium chloride methanol monosolvate Atomic displacement parameters (Å^2^)

**Table:**

	*U*^11^	*U*^22^	*U*^33^	*U*^12^	*U*^13^	*U*^23^
Cl1	0.0203 (4)	0.0328 (4)	0.0450 (5)	−0.0020 (2)	0.0147 (3)	−0.0022 (3)
Cl2	0.0257 (4)	0.0235 (4)	0.0255 (4)	−0.0107 (2)	0.0027 (3)	−0.0020 (2)
Cl3	0.0208 (4)	0.0355 (4)	0.0181 (4)	0.0118 (2)	−0.0009 (3)	0.0001 (2)
O1	0.0188 (9)	0.0194 (8)	0.0212 (9)	−0.0057 (6)	−0.0002 (7)	0.0054 (7)
N1	0.0119 (10)	0.0142 (9)	0.0156 (10)	−0.0010 (7)	0.0055 (8)	0.0012 (7)
N2	0.0084 (9)	0.0168 (9)	0.0140 (10)	0.0003 (7)	0.0051 (8)	−0.0007 (7)
C1	0.0128 (11)	0.0129 (9)	0.0161 (11)	0.0003 (8)	0.0045 (9)	−0.0005 (8)
C2	0.0185 (12)	0.0207 (11)	0.0146 (11)	−0.0040 (9)	0.0024 (10)	0.0013 (9)
C3	0.0175 (12)	0.0200 (11)	0.0115 (11)	−0.0015 (9)	0.0008 (9)	0.0027 (9)
C4	0.0210 (13)	0.0181 (11)	0.0174 (11)	−0.0008 (9)	0.0060 (10)	−0.0001 (9)
C5	0.0185 (13)	0.0220 (11)	0.0184 (12)	0.0003 (10)	0.0053 (10)	0.0032 (9)
C6	0.0198 (13)	0.0202 (11)	0.0173 (12)	−0.0071 (9)	0.0011 (10)	−0.0011 (9)
C7	0.0250 (14)	0.0225 (11)	0.0230 (13)	−0.0016 (10)	0.0065 (11)	−0.0064 (10)
C8	0.0193 (13)	0.0254 (12)	0.0203 (12)	−0.0013 (10)	0.0063 (10)	−0.0034 (10)
C9	0.0197 (12)	0.0218 (11)	0.0175 (12)	−0.0088 (9)	0.0054 (10)	0.0027 (9)
C10	0.0095 (11)	0.0162 (10)	0.0161 (11)	0.0017 (8)	0.0033 (9)	0.0015 (8)
C11	0.0052 (10)	0.0160 (10)	0.0160 (11)	−0.0005 (8)	0.0051 (9)	0.0006 (8)
C12	0.0089 (11)	0.0220 (11)	0.0164 (11)	−0.0004 (9)	0.0029 (9)	0.0004 (9)
C13	0.0075 (11)	0.0239 (11)	0.0216 (12)	−0.0005 (9)	0.0026 (9)	0.0018 (9)
C14	0.0082 (11)	0.0284 (12)	0.0290 (14)	0.0036 (9)	0.0048 (10)	0.0002 (11)
C15	0.0118 (12)	0.0229 (11)	0.0210 (12)	0.0046 (9)	0.0076 (10)	−0.0025 (9)
C16	0.0099 (11)	0.0220 (11)	0.0239 (13)	−0.0038 (9)	0.0076 (9)	0.0054 (9)
C17	0.0112 (11)	0.0384 (14)	0.0188 (12)	0.0005 (10)	0.0076 (10)	0.0070 (11)
C18	0.0124 (12)	0.0364 (14)	0.0204 (13)	0.0057 (10)	0.0079 (10)	−0.0014 (10)
C19	0.0112 (11)	0.0200 (11)	0.0174 (11)	0.0014 (9)	0.0064 (9)	−0.0042 (9)
O2	0.0258 (11)	0.0346 (10)	0.0648 (15)	0.0065 (9)	0.0277 (10)	0.0167 (11)
C20	0.0235 (14)	0.0398 (15)	0.0308 (15)	0.0044 (11)	0.0121 (12)	0.0006 (12)

### 1-{2-[2-(3,4-Dichlorophenyl)-N-methylacetamido]cyclohexyl}pyrrolidin-1-ium chloride methanol monosolvate Geometric parameters (Å, º)

**Table:**

Cl1—C5	1.734 (3)	C11—H11	1.0000
Cl2—C6	1.735 (2)	C11—C12	1.532 (3)
O1—C1	1.228 (3)	C12—H12A	0.9900
N1—C1	1.363 (3)	C12—H12B	0.9900
N1—C9	1.461 (3)	C12—C13	1.531 (3)
N1—C10	1.471 (3)	C13—H13A	0.9900
N2—H2	1.0000	C13—H13B	0.9900
N2—C11	1.511 (3)	C13—C14	1.522 (3)
N2—C16	1.527 (3)	C14—H14A	0.9900
N2—C19	1.520 (3)	C14—H14B	0.9900
C1—C2	1.526 (3)	C14—C15	1.530 (3)
C2—H2A	0.9900	C15—H15A	0.9900
C2—H2B	0.9900	C15—H15B	0.9900
C2—C3	1.502 (3)	C16—H16A	0.9900
C3—C4	1.386 (3)	C16—H16B	0.9900
C3—C8	1.389 (3)	C16—C17	1.519 (4)
C4—H4	0.9500	C17—H17A	0.9900
C4—C5	1.390 (4)	C17—H17B	0.9900
C5—C6	1.389 (3)	C17—C18	1.527 (4)
C6—C7	1.384 (4)	C18—H18A	0.9900
C7—H7	0.9500	C18—H18B	0.9900
C7—C8	1.387 (4)	C18—C19	1.514 (3)
C8—H8	0.9500	C19—H19A	0.9900
C9—H9A	0.9800	C19—H19B	0.9900
C9—H9B	0.9800	O2—H2C	0.8400
C9—H9C	0.9800	O2—C20	1.400 (4)
C10—H10	1.0000	C20—H20A	0.9800
C10—C11	1.536 (3)	C20—H20B	0.9800
C10—C15	1.529 (3)	C20—H20C	0.9800
			
C1—N1—C9	121.69 (19)	C11—C12—H12B	109.8
C1—N1—C10	118.92 (18)	H12A—C12—H12B	108.3
C9—N1—C10	119.25 (18)	C13—C12—C11	109.25 (19)
C11—N2—H2	106.9	C13—C12—H12A	109.8
C11—N2—C16	115.89 (16)	C13—C12—H12B	109.8
C11—N2—C19	112.93 (16)	C12—C13—H13A	109.6
C16—N2—H2	106.9	C12—C13—H13B	109.6
C19—N2—H2	106.9	H13A—C13—H13B	108.1
C19—N2—C16	106.78 (17)	C14—C13—C12	110.41 (19)
O1—C1—N1	122.7 (2)	C14—C13—H13A	109.6
O1—C1—C2	121.3 (2)	C14—C13—H13B	109.6
N1—C1—C2	115.95 (19)	C13—C14—H14A	109.6
C1—C2—H2A	109.3	C13—C14—H14B	109.6
C1—C2—H2B	109.3	C13—C14—C15	110.1 (2)
H2A—C2—H2B	107.9	H14A—C14—H14B	108.1
C3—C2—C1	111.69 (18)	C15—C14—H14A	109.6
C3—C2—H2A	109.3	C15—C14—H14B	109.6
C3—C2—H2B	109.3	C10—C15—C14	111.43 (19)
C4—C3—C2	120.5 (2)	C10—C15—H15A	109.3
C4—C3—C8	119.1 (2)	C10—C15—H15B	109.3
C8—C3—C2	120.4 (2)	C14—C15—H15A	109.3
C3—C4—H4	119.9	C14—C15—H15B	109.3
C3—C4—C5	120.2 (2)	H15A—C15—H15B	108.0
C5—C4—H4	119.9	N2—C16—H16A	110.9
C4—C5—Cl1	119.08 (19)	N2—C16—H16B	110.9
C6—C5—Cl1	120.65 (19)	H16A—C16—H16B	108.9
C6—C5—C4	120.3 (2)	C17—C16—N2	104.37 (18)
C5—C6—Cl2	121.0 (2)	C17—C16—H16A	110.9
C7—C6—Cl2	119.16 (19)	C17—C16—H16B	110.9
C7—C6—C5	119.9 (2)	C16—C17—H17A	111.2
C6—C7—H7	120.2	C16—C17—H17B	111.2
C6—C7—C8	119.6 (2)	C16—C17—C18	103.0 (2)
C8—C7—H7	120.2	H17A—C17—H17B	109.1
C3—C8—H8	119.5	C18—C17—H17A	111.2
C7—C8—C3	121.0 (2)	C18—C17—H17B	111.2
C7—C8—H8	119.5	C17—C18—H18A	111.3
N1—C9—H9A	109.5	C17—C18—H18B	111.3
N1—C9—H9B	109.5	H18A—C18—H18B	109.2
N1—C9—H9C	109.5	C19—C18—C17	102.46 (19)
H9A—C9—H9B	109.5	C19—C18—H18A	111.3
H9A—C9—H9C	109.5	C19—C18—H18B	111.3
H9B—C9—H9C	109.5	N2—C19—H19A	110.7
N1—C10—H10	107.6	N2—C19—H19B	110.7
N1—C10—C11	111.59 (17)	C18—C19—N2	105.39 (19)
N1—C10—C15	111.59 (18)	C18—C19—H19A	110.7
C11—C10—H10	107.6	C18—C19—H19B	110.7
C15—C10—H10	107.6	H19A—C19—H19B	108.8
C15—C10—C11	110.56 (18)	C20—O2—H2C	109.5
N2—C11—C10	110.15 (17)	O2—C20—H20A	109.5
N2—C11—H11	107.8	O2—C20—H20B	109.5
N2—C11—C12	112.64 (17)	O2—C20—H20C	109.5
C10—C11—H11	107.8	H20A—C20—H20B	109.5
C12—C11—C10	110.44 (17)	H20A—C20—H20C	109.5
C12—C11—H11	107.8	H20B—C20—H20C	109.5
C11—C12—H12A	109.8		
			
Cl1—C5—C6—Cl2	−0.9 (3)	C9—N1—C1—O1	−178.5 (2)
Cl1—C5—C6—C7	−179.39 (19)	C9—N1—C1—C2	1.3 (3)
Cl2—C6—C7—C8	−179.4 (2)	C9—N1—C10—C11	67.1 (2)
O1—C1—C2—C3	−5.6 (3)	C9—N1—C10—C15	−57.1 (3)
N1—C1—C2—C3	174.6 (2)	C10—N1—C1—O1	5.8 (3)
N1—C10—C11—N2	53.5 (2)	C10—N1—C1—C2	−174.32 (18)
N1—C10—C11—C12	178.53 (17)	C10—C11—C12—C13	58.9 (2)
N1—C10—C15—C14	−179.97 (18)	C11—N2—C16—C17	112.6 (2)
N2—C11—C12—C13	−177.43 (16)	C11—N2—C19—C18	−140.93 (19)
N2—C16—C17—C18	35.0 (2)	C11—C10—C15—C14	55.2 (2)
C1—N1—C10—C11	−117.1 (2)	C11—C12—C13—C14	−60.3 (2)
C1—N1—C10—C15	118.6 (2)	C12—C13—C14—C15	58.8 (3)
C1—C2—C3—C4	90.5 (3)	C13—C14—C15—C10	−56.2 (3)
C1—C2—C3—C8	−87.8 (3)	C15—C10—C11—N2	178.31 (17)
C2—C3—C4—C5	−178.5 (2)	C15—C10—C11—C12	−56.6 (2)
C2—C3—C8—C7	177.3 (2)	C16—N2—C11—C10	69.3 (2)
C3—C4—C5—Cl1	179.93 (18)	C16—N2—C11—C12	−54.5 (2)
C3—C4—C5—C6	0.9 (4)	C16—N2—C19—C18	−12.4 (2)
C4—C3—C8—C7	−1.1 (4)	C16—C17—C18—C19	−42.8 (2)
C4—C5—C6—Cl2	178.10 (19)	C17—C18—C19—N2	33.9 (2)
C4—C5—C6—C7	−0.4 (4)	C19—N2—C11—C10	−167.08 (17)
C5—C6—C7—C8	−0.9 (4)	C19—N2—C11—C12	69.1 (2)
C6—C7—C8—C3	1.6 (4)	C19—N2—C16—C17	−14.1 (2)
C8—C3—C4—C5	−0.2 (3)		

### 1-{2-[2-(3,4-Dichlorophenyl)-N-methylacetamido]cyclohexyl}pyrrolidin-1-ium chloride methanol monosolvate Hydrogen-bond geometry (Å, º)

**Table:**

*D*—H···*A*	*D*—H	H···*A*	*D*···*A*	*D*—H···*A*
N2—H2···Cl3	1.00	2.17	3.0775 (19)	150
C2—H2*A*···Cl3	0.99	2.75	3.486 (2)	132
C10—H10···O1^i^	1.00	2.61	3.543 (3)	156
C16—H16*B*···O1^i^	0.99	2.29	3.274 (3)	170
C19—H19*A*···Cl3^ii^	0.99	2.92	3.685 (2)	135
O2—H2*C*···Cl3	0.84	2.32	3.103 (2)	155

Symmetry codes: (i) −*x*+2, −*y*+1, −*z*+1; (ii) *x*+1/2, −*y*+3/2, *z*+1/2.
